# Compound Glass Microsphere Resonator Devices

**DOI:** 10.3390/mi9070356

**Published:** 2018-07-19

**Authors:** Jibo Yu, Elfed Lewis, Gerald Farrell, Pengfei Wang

**Affiliations:** 1 Key Laboratory of In-fiber Integrated Optics of the Ministry of Education, College of Science, Harbin Engineering University, Harbin 150001, China; yu20131164@hrbeu.edu.cn; 2 Optical Fibre Sensors Research Centre, Department of Electronic and Computer Engineering, University of Limerick, Limerick V94 T9PX, Ireland; Elfed.Lewis@ul.ie; 3 Photonics Research Centre, Dublin Institute of Technology, Kevin Street, 8 Dublin D08 NF82, Ireland; gerald.farrell@dit.ie; 4 Key Laboratory of Optoelectronic Devices and Systems of Ministry of Education and Guangdong Province, College of Optoelectronic Engineering, Shenzhen University, Shenzhen 518060, China

**Keywords:** compound glass, microsphere, resonator, lasing, sensing

## Abstract

In recent years, compound glass microsphere resonator devices have attracted increasing interest and have been widely used in sensing, microsphere lasers, and nonlinear optics. Compared with traditional silica resonators, compound glass microsphere resonators have many significant and attractive properties, such as high-Q factor, an ability to achieve high rare earth ion, wide infrared transmittance, and low phonon energy. This review provides a summary and a critical assessment of the fabrication and the optical characterization of compound glasses and the related fabrication and applications of compound glass microsphere resonators.

## 1. Introduction

Over the past few decades, research interest in microsphere resonators has grown rapidly. For a microsphere resonator, pump light is coupled into the microsphere through a tapered optical fiber or via free space. The coupled light signal is totally internally reflected and contained within the microsphere cavity to provide a ‘whispering-gallery mode’ (WGM) light resonance. Because of its extremely high-Q and small mode volume, microsphere resonators have many important roles in both active and passive photonic devices, such as in optical feedback, non-linear optics, low threshold lasers, dispersion managed optical systems, and energy storage [1,2,3,4,5,6,7].

Most current microsphere resonators are fabricated by melting the tip of a fused silica optical fiber [8], but is also possible to fabricate microsphere resonator from compound glass materials other than silica. Different host materials have different physical-chemical properties when in the form of compound glass, and many optical phenomena are limited by the properties of the material, therefore microspheres made from compound glass can behave differently in different practical applications. For example, glass materials with a high nonlinear coefficient can be used in wavelength conversion, optical switching and signal regeneration; high rare-earth ion doped glass materials have wide applications in near Near-infrared (NIR) and Mid-infrared (MIR) lasers, and some glasses are sensitive to temperature, light, and greenhouse gases. Such materials can be used to fabricate many different compound glass microsphere sensors [9,10,11,12,13].

In this paper, the progress of compound glass based microsphere-resonator devices over the past few decades is discussed. In the first section, the properties of the various compound glass materials are introduced, where the glass materials are divided into conventional glass and heavy metal glass types. In the second section, the fabrication methods for conventional silica microspheres and compound glass microspheres are reviewed. Finally, the applications of compound glass microsphere in microcavity lasing, nonlinear optical phenomena, and optical sensing are discussed, and the characterization of some compound glass microspheres with high-Q resonance are also presented.

## 2. Glass Materials

Oxide glasses have good chemical stability and excellent mechanical properties. Compared with metals, oxide glass materials have lower cost and typically possess an immunity to electromagnetic interference. Therefore, practical applications have focused on oxide glasses. Oxide glasses are generally divided into conventional oxide glasses and heavy metal oxide glasses. Conventional oxide glasses usually include silicate, germanate, and phosphate glasses, while heavy metal oxides usually comprise lead silicate, tellurite, and bismuth glasses. While the former have better thermal and chemical stability, the latter involve relatively simple fabrication processes as they can be processed at lower temperatures.

Heavy metal oxide glasses are predominantly used as a MIR material as they have excellent prospects for future developments in this wavelength domain e.g., Gas Sensing. They are principally based on PbO, TeO, and BiO. Heavy metal oxide glasses have many attractive optical properties, including high density, high refractive index, and excellent infrared transmission, for which the infrared wavelength band offers a broad range of applications. Compared with conventional oxide glasses, they have lower phonon energy and a broader infrared transmission range [14]. The low phonon energy in glasses reduces the relaxation rate of multiple phonons and therefore increases the probability of radiative transitions [15,16,17].

Heavy metal oxide glasses have better physical-chemical characteristics and preparation processes than chalcogenide glasses, while chalcogenide glasses have lower phonon energy and probability of multiple phonon relaxation compared with oxide glasses; chalcogenide glasses are also very suitable as host materials for rare earth doping. In particular, chalcogenide glasses have a wide infrared transmission window, and have received much attention as a material for MIR emission [18,19,20,21,22].

The physical-chemical properties of conventional oxide glasses, heavy metal oxide glasses, and chalcogenide glasses are introduced in detail in the following sub-sections, and are pinned to their related application areas where appropriate.

### 2.1. Conventional Oxide Glass

#### 2.1.1. Silicate Glass

The main components of silicate glasses tend to be SiO_2_-Al_2_O_3_-R_2_O, SiO_2_-P_2_O_5_-R_2_O, SiO_2_-B_2_O_3_-R_2_O, SiO_2_-GeO_2_-R_2_O, or a mix of the above, with a high SiO_2_ content and relatively a low R_2_O content. The glass often exhibits a small expansion coefficient, small dispersion, as well as excellent chemical and thermal stability. Silicate glasses are often used as optical glasses, in solar panels, liquid crystal display substrates, and heat collectors and silicate glasses have also been used in blue-violet LEDs to provide a new lighting sources [23]. In addition, it is worth noting that for the application of this glass in solar panels, high energy photon cutting can be achieved by rare-earth ions doping, which can significantly improve solar cell conversion efficiency [5,24].

The silicon-oxygen tetrahedron network of the silicate glass provides the excellent macroscopic mechanical and chemical stability of the material. When the silicate glass is melted in the fabrication process, the resulting loss of glass is relatively small, but a high melting temperature is required.

At present, active and passive optical fibers based on quartz materials are widely used, especially in high-power fiber lasers [25,26]. Quartz optical fibers exhibit low transmission loss, have a high thermal damage threshold, high mechanical strength and a high resistance to bending. However, quartz glass also has significant shortcomings. The silicon oxygen network of the quartz glass cannot provide enough non-bridging oxygens, which makes it very easy for rare earth ions to produce clusters, which leads to undesirable fluorescence quenching. In practical applications, the background loss of the optical fiber needs to be controlled to a very low value, and the introduction of other metal ions should be avoided as much as possible. Therefore, there is a pressing need to find a new way to increase the solubility of rare earth ions in silica glasses.

#### 2.1.2. Phosphate Glass

Doping of rare earth ions in the phosphate glass can result in the fabricated phosphate fiber having the attractive characteristics of short length, small volume, high energy conversion efficiency, and low cost for fiber lasers, arousing the interest of many researchers [27,28,29,30,31]. Phosphate glasses have a higher rare earth ion solubility compared with silicate glasses, up to 10^26^ ions/m^3^, so they can be doped with high concentrations of rare earth ions to obtain higher gains [32,33]. Furthermore, rare earth doped phosphate glass fibers can achieve the required gain within a few centimeters, avoiding the disadvantages of unwanted nonlinear phenomena associated with the long lengths of quartz fibers.

The basic structural unit of P_2_O_5_ glass is a phosphorus-oxygen tetrahedron [PO_4_], where the phosphorus atom bonds to each oxygen atom with a double bond [34]. However, all the basic structural polyhedrons of tellurite glass and silicate glasses are connected by bridging oxygens, while the phosphorus tetrahedrons with double bonds lead to asymmetry in the glass structure of P_2_O_5_, resulting in a low viscosity, a large thermal expansion coefficient, and a poor chemical stability. In spite of these disadvantages, phosphate glasses still possesses many advantageous characteristics, such as a long fluorescence lifetime, large stimulated emission cross section, large gain coefficient, moderate phonon energy, and low fluorescence quenching.

### 2.2. Low-Phonon-Energy Oxide Glass

#### 2.2.1. Germanate and Germanosilicate Glass

Germanate glass is a heavy metal oxide glass, with a wide infrared transmission window (~6 μm) and low phonon energy (~850 cm^−1^), making it an ideal candidate material in for use in the MIR wavelength region [35]. Compared with fluoride and chalcogenide glasses, germanate glasses have good thermal stability, a simple fabrication process, and superior mechanical properties, leading to greater robustness and stability [36].

Conventional silicate fibers have excellent chemical stability and mechanical properties and their good plasticity enables fabrication into a variety of shapes, such as rods, plates, and optical fiber. This type of glass is therefore currently used in a wide variety of optical materials. However, the silicate glass material has a high phonon energy, resulting in an increase in the probability of non-radiative transitions, which limits the application of silicate glasses in photonics. Germanate glass have a lower phonon energy, which is crucial to increasing the radiative transition rate and probability of infrared transmission of rare earth ions and thus is a better material for obtaining high luminosity in the infrared band. However, high purity germanate glasses are extremely expensive, so only a limited amount of research has been conducted on this material [37].

Both silicon and germanium are in the periodic table group IVA and the outermost electron layer structure is in the form of ns^2^np^2^. The same main group elements have many similar chemical and physical properties. Silicon and germanium exist in the form of tetrahedral structure [SiO_4_] and [GeO_4_], thus the partial replacement of silicon oxide with germanium oxide in silicate glasses not only retains the excellent physical-chemical properties of the silicate glass, but also reduces the viscosity and fusion temperature of the glass, and improves the solubility of rare earth ions.

Germanosilicate glasses have received a lot of attention because they make up for the lack of development and utilization of germanate and silicate glasses in the optical field, which has resulted in germanium silicate glass having many applications in optical fiber communications, military detection and lasers.

#### 2.2.2. Tellurite Glass

Early in 1952, Stanworth J. had studied the formation and structure of tellurite glasses [38]. However, the TeO_2_ raw material was expensive, and hence tellurite glasses were considered to be of low practical value and had not been pursued as a candidate optical material until 1994. In this year, Wang. J. S of Rutgers University studied the tellurite glass as an optical material and found that it had a high rare earth ion solubility and constituted a new type of glass that could be used in optical fiber devices. Shortly afterwards, the Nd^3+^ doped tellurite fiber was fabricated and used to demonstrate single-mode laser output [39]. In 1997, Japan’s NTT company successfully prepared an erbium doped fiber that could be used for broadband amplifiers, and quickly inspired many scientists to research tellurite glasses [40,41].

The phonon energy in tellurite glasses is low, generally in the range 650–800 cm^−1^. The low non-radiative transition rate enhances the luminescence of the glass in the infrared. In addition, the maximum phonon energy of the tellurite glass system is the closest to that of fluoride glass, and the tellurite glass is most likely to replace fluoride glass as a host material, making it capable of forming a laser output in mid-infrared wavelength band.

Tellurite glass has a higher rare earth ion solubility than silica, which can be attributed to the fact that the rare earth ions in the tellurite glass can replace the position of the network modifier, which weakens the clustering phenomenon in the tellurite glass. Compared with fluoride glasses, they have good chemical stability, thermal stability, and mechanical properties and can also be fabricated using a relatively simple process. The melting temperature of tellurite glasses is generally around 800 °C. Tellurite glass has a high refractive index (1.8–2.3) compared to fluoride (~1.4), germanate glass (~1.6), and quartz (~1.45) and different structural units, such as [TeO_4_], [TeO_3_], and [TeO_3_^+1^] [14]: this useful diversity of structural units can provide a more coordinated field environment for rare earth ions, thus the quenching phenomenon in tellurite glasses is greatly reduced.

#### 2.2.3. Bismuth Glass

The electronic layer structure of Bi is [Xe]4f^14^5d^10^6s^2^6p^3^, and this element has been widely studied. Since the electrons of the p orbital are easily involved in chemical bonding, Bi is also known as “The Wonder Metal”. There are three distinct properties of the Bi element. Firstly, Bi has many valence states, such as 0, +1, +2, +3, and +5, so there are many states of matter, and the electrons of Bi element in the 6p, 6s, and 5d orbitals are very sensitive to the surrounding environment. Secondly, Bi has a strong cluster phenomenon, which is widely found in molten Lewis acids, molecular crystals, or porous zeolite solids. Thirdly, Bi shows a strong spin-orbit coupling effect, which allows them to act as optically active centers in different host materials. Bi doped materials exhibit a rich luminescent characteristic, which makes them different from the conventional active centers such as lanthanides and transition metals.

Bismuth glass has many advantages including low phonon energy (circa 600 cm^−1^), high refractive index (circa 1.9), a wide infrared transmission range (0.4–6.5 μm), strong corrosion resistance, good solubility of rare earth ions, relatively good chemical stability, and low material cost, which results in many applications in photonics.

On one hand, bismuth glass is considered an innovative host material, and it is capable of providing efficient up conversion to red and green light output. On the other hand, bismuth glass is one of the most promising gain media materials currently used for rare earth doped fiber amplifiers. Many reports have shown that the host materials of bismuth glass have good performance in high capacity and ultra-wideband communications [42,43,44].

#### 2.2.4. Lead Silicate Glass

Heavy metal silicate glasses have attracted wide attention in the field of photonic crystal fibers, because the addition of heavy metal ions that can increase the luminescent properties of the glass and change its nonlinear coefficient [44]. The nonlinear coefficient n_2_ of lead silicate glasses is more than 20 times that of quartz, and it has the advantages of low melting temperature, moderate phonon energy (955 cm^−1^), high rare earth ion doping ability, and a large emission cross section, making it an ideal choice for manufacturing photonic crystal fibers.

Lead silicate glass is a mature optical host material, which has been applied in lasers, military, construction, medical and other areas, as well as its application as a gain medium in laser and amplifier systems. Lead silicate glass has a high damage threshold, better resistance to crystallization, and greater mechanical strength. When added to conventional quartz fibers, Pb loosens the network structure of quartz, which accepts a high concentration of dopant ions and results in a suitably low fiber drawing temperature and could result in higher power laser outputs [45].

### 2.3. Chalcogenide Glass

Research on the optical properties of chalcogenide glass started nearly 60 years ago [46]. Chalcogenide glasses are composed of heavy elements joined by covalent bonds, resulting in unique optical properties making it highly suitable as a material for use in the MIR region, nonlinear optics, and optical waveguides. The emission of the chalcogenide glass red shifts to the visible or MIR region of the spectrum because the interatomic bond energy in chalcogenide glass is weaker than that in the oxide glass case. As the constituent atoms are heavier, the energy of the bond energy is very low, which means chalcogenide glass is transparent in the MIR region, and the low phonon energy (550 cm^−1^) makes it an excellent host material for rare earth doping [47].

In general, the infrared transmission of chalcogenide glass extends up to 11 μm, with selenides reaching up to 15 μm and tellurite exceeding 20 μm. However, the physical properties of the glass transition temperature, hardness, strength, and durability usually degrade with weaker valence bonds, narrowing the long wave transparency band.

The low transition temperature means that precision glass forming offers a viable solution for the manufacture of low cost optical components such as those used in thermal imaging [48]. Chalcogenide glasses have a high refractive index, up to 2 to 3. According to Miller’s formula [49], the higher the refractive index of a material, the higher its nonlinear coefficient n_2_. As a consequence, the third-order Kerr effect of chalcogenide is several thousand times higher than that of silica [50,51,52], which means that chalcogenide glasses are considered excellent media for all-optical signal processing [43].

Chalcogenide glasses are also photosensitive. When exposed near the energy band, the chemical bond energy changes [53], and similar changes occur under heating and exposure to X-rays and electron beams. The inherent transparent window of chalcogenide glass is mainly in the molecular fingerprint region of 2–25 μm, making chalcogenide glass suitable for use in MIR optical fiber transmission, optical sensing, and as a waveguide material in optical communication.

Chalcogenide glass fibers were first reported in 1980 [54], when it was found that the limitation of high transmission loss was mainly due to impurity absorption. The relatively high loss of chalcogenides is still a significant problem, limiting its use to short lengths of fiber. Chalcogenide glasses require a high degree of purification during processing regardless of the final application. The high purity chalcogenide glass materials which do not include distillation in their processing often include oxygen, carbon, and hydrogen impurities [55,56], and these result in strong absorption peaks which occur within the 1.4–14.9 μm band.

There are many ways to reduce the impurities in chalcogenide glasses, which include: removing surface oxides in vacuum; chemical distillation using oxygen sorbents; treatment with thorium halide or active chlorine; evaporation through a porous silica frit; dynamic pyrolysis and purification of chalcogenide by high temperature oxidation [57,58,59,60,61]. These methods can reduce the impurity content to 10–5%, which significantly increases the infrared transparency. 

## 3. Fabrication of Compound Glass Microspheres

At present, the principal method used for making microsphere resonators is based on melting of the glass materials. The optical loss factor of organic materials is generally large, and it is difficult to obtain a high-Q microsphere resonator, thus most microspheres are made from glass. The melting method uses the surface tension of molten glass to fabricate microspheres. In the case of a quartz fiber microspheres, fabrication usually involves the use of a CO_2_ laser or heating furnace in their preparation. For other compound glass materials, there are slight differences in the preparation process. In this section, the manufacturing methods of traditional silica microspheres and compound glass microspheres are discussed.

### 3.1. Fabrication of Silica Microspheres

Low-cost standard single-mode fiber is widely used to produce high-Q silica microsphere resonators. The optical fiber generally used in the fabrication process is SMF-28 single-mode fiber (Corning), and its transmission loss in the telecommunication window is less than 0.2 dB/km. The diameters of the core and cladding are 8.2 μm and 125 μm, respectively.

**Figure 1:**
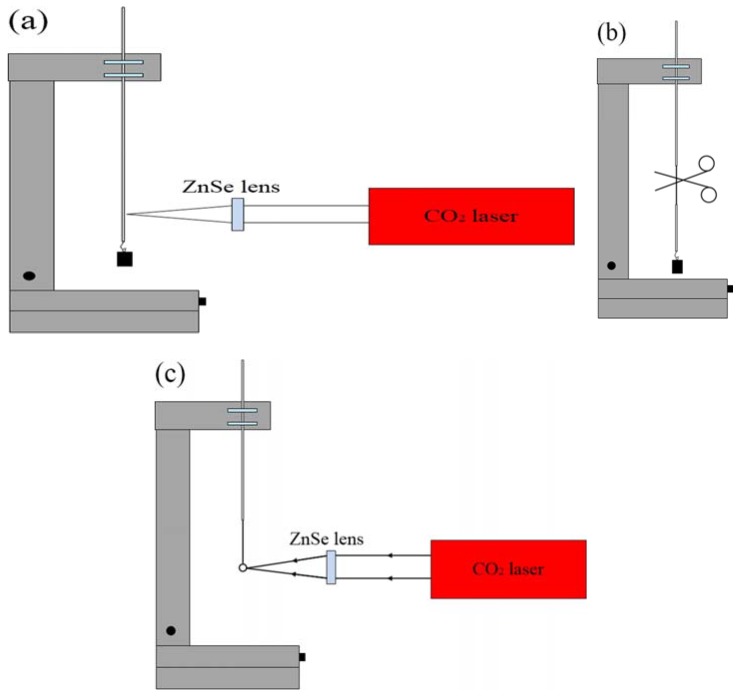
Schematic diagram of the experimental setup for making a silica microsphere. (**a**) A ZnSe lens is used to focus a CO_2_ laser beam on the silica fiber; (**b**) the waist region of tapered fiber is cleaved; (**c**) a silica microsphere is obtained by focusing a CO_2_ laser beam on the end of the cleaved tapered fiber.

**Figure 2:**
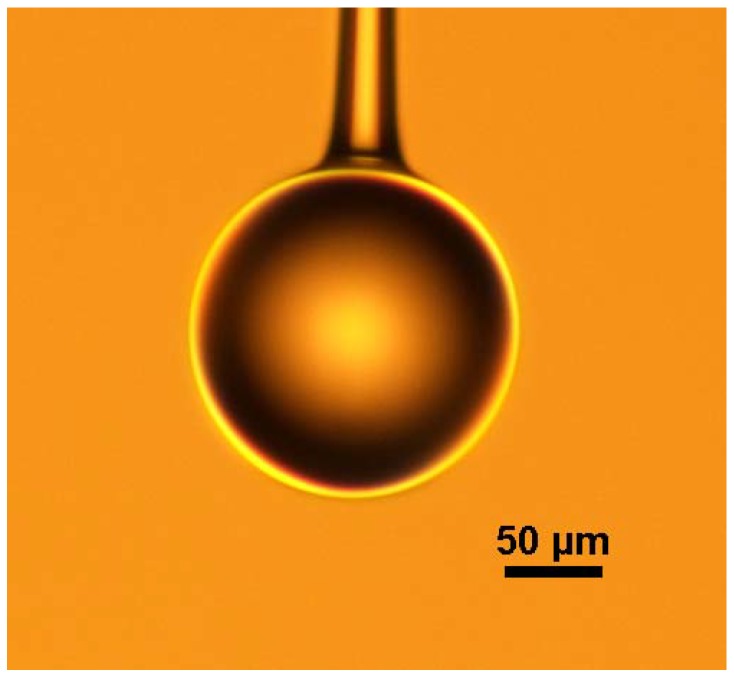
Microscope image of silica microsphere made with CO_2_ laser.

The schematic diagram of the experimental setup for making a microsphere resonator is shown in Figure 1. The main instrument used in the experiment is a precision three-dimensional (3D) translation stage, a continuous CO_2_ laser with a wavelength of 10.6 μm and a ZnSe lens for focusing. The experimental step of fabricating the silica microsphere resonator can be divided into three stages. In the first step, the coating layer at the end of the single-mode is removed, the fiber is mounted vertically on the 3D translation stage, and a weight is hung at the end of the fiber. Using a ZnSe lens to focus the laser beam on the single-mode fiber, the fiber absorbs light, resulting in a temperature rise. The glass softens and gradually turns into a tapered fiber under the influence of the weight. The heating is terminated when the waist diameter of the tapered fiber reaches around 100 μm. In the second step, the tapered fiber is accurately cleaved at the waist region to obtain a half tapered fiber. In the third step, using a ZnSe lens once more to focus the laser beam on the end of the half tapered fiber, the silica microsphere is formed at the fiber end due to the surface tension acting on the molten glass. The microscope image of a silica microspheres fabricated in this manner is shown in Figure 2.

In addition to employing the method referred to above, another commonly method for making silica microsphere is based on arc discharge [62,63]. In the literature [63], a fabrication process for silica microspheres using a Fitel S182PM fusion splicer was introduced. Firstly, the coating layer of the SMF28 fiber was removed, then the electrode of the splice was used to discharge the tip of the fiber, and the silica microsphere was formed due to the surface tension. In the experiment, the arc power was 110 a.u., the premelting time was 240 ms, and the arc duration was 2000 ms.

### 3.2. Fabrication of Lead Silicate Microspheres

In this section, a method of fabricating a lead silicate microspheres is introduced [64], and is similar to the method that uses a CO_2_ laser to fabricate a silica optical microsphere as described in the previous section. The glass fiber is softened using a heat source and then the microspheres are formed due to surface tension of the glass fiber.

**Figure 3:**
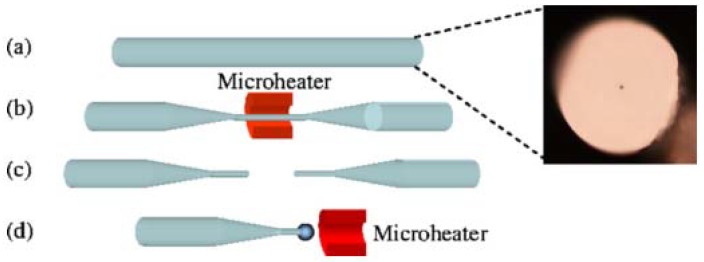
Schematic diagram of the experimental setup used for making a lead silicate microsphere. (**a**) Cross-section of lead silicate fiber; (**b**) a fiber is tapered by using a microheater; (**c**) the uniform waist of the tapered fiber is cleaved; (**d**) a lead silicate microsphere is formed. Reprinted/Adapted with permission from [64].

The schematic diagram of the experimental setup for making a lead silicate microsphere is shown in Figure 3. First of all, the lead silicate glass fiber is tapered to make the diameter of the lead silicate glass microsphere much smaller than the outside diameter of the lead silicate glass fiber. Then, the tapered section is placed in a resistive microheater with a Ω-shaped opening and heated to circa 500 °C. The microheater is moved back and forth along the fiber, while both ends of the fiber are carefully drawn using a computer-controlled translation stage. In this way, a tapered fiber of uniform waist diameter (d < 10 μm) and transition region are obtained [65]. The tapered fiber is then cut at the middle of the uniform waist. The newly formed half tapered fiber is then positioned in close proximity to the microheater and heated to 900 °C, which is higher than the softening point of the glass and thus the fused lead silicate glass fiber is melted to form the microsphere due to the surface tension of the lead silicate glass fiber.

### 3.3. Fabrication of Germanium Microspheres

An experimental method for fabricating germanium microspheres using a germanium glass optical fiber is described in [66]. The material of the glass cladding is borosilicate, and the core is germanium. The core and the cladding diameters are typically 15 μm and 150 μm [67], respectively.

In order to obtain a germanium microsphere, the first step was to heat the core of the germanium with the fabrication method outlined for the quartz microsphere mentioned above, focusing the CO_2_ laser beam on the core of the germanium glass. It is worth noting that when the temperature was between the melting point (938 °C) of the core and the softening point (1260 °C) of the cladding, a germanium microsphere would be formed inside the fiber cladding due to the lower cladding viscosity, which results in excellent smoothness for the glass surface.

**Figure 4:**
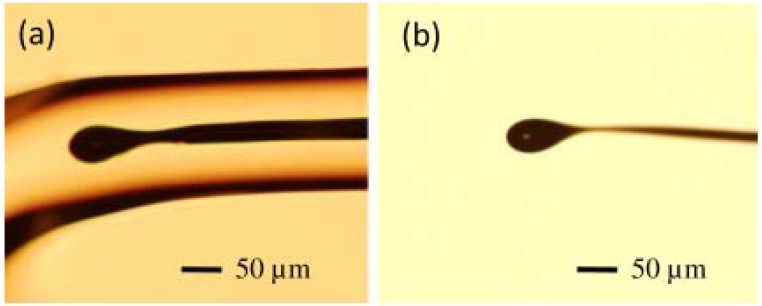
Microscope image of (**a**) a germanium microsphere in borosilicate cladding and (**b**) a fabricated germanium microsphere. Reprinted/Adapted with permission from [66].

The diameter of the resulting microsphere was 40–50 μm, and Figure 4a shows the microscope image of the microsphere embedded in a borosilicate cladding. The majority of the glass cladding was then etched away using a 48% HF solution, followed by immersion of the microsphere in a mixture of ammonium fluoride buffer and HF (20:1) for 5–10 min. Finally, the residual HF solution on the surface of the microsphere was cleaned with deionized water. The microscope image of the resulting germanium microsphere is shown in Figure 4b.

### 3.4. Fabrication of Chalcogenide Microspheres

There have been many reports on the fabrication methods for chalcogenide glass microspheres [22,68,69,70,71]. One of them is a relatively simple method involving the use of a ceramic microheater to pull apart the molten chalcogenide glass [70]. This is possible due to the low softening temperature (100–400 °C) and transition temperature (100–300 °C) of chalcogenide glasses.

**Figure 5:**
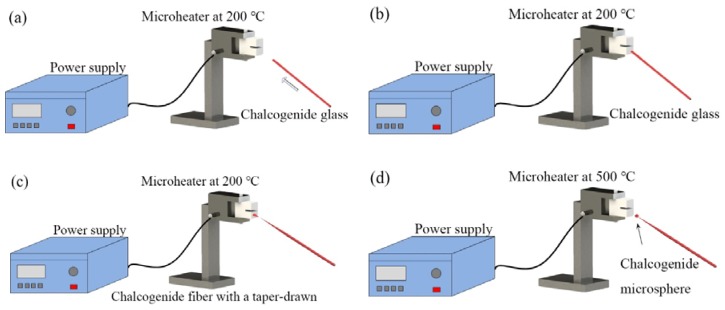
Experimental step for fabricating a chalcogenide microsphere. (**a**) Chalcogenide glass is moved to a microheater at 200 °C; (**b**) the chalcogenide glass is attached on the surface of microheater; (**c**) the chalcogenide glass is drawn to a half tapered fiber; (**d**) a chalcogenide microsphere is formed.

The chalcogenide glass fiber used in the experiment was a commercial step multimode fiber provided by Oxford Electronics. The core and cladding materials are As_2_S_3_ and As_x_S_1−x_, with diameters of 180 μm and 275 μm, respectively. The experimental step for fabricating the chalcogenide glass microsphere is shown in Figure 5. At first, the ceramic microheater was heated to about 200 °C, and a chalcogenide glass fiber was moved in close proximity to the microheater. Then, the end of the chalcogenide fiber was placed in contact with the outer wall of the ceramic microheater and as a result, the end of the glass fiber softened. Next, the fiber was pulled at the speed of 0.1–1 m/s until the end of the glass fiber was broken, resulting in a long half tapered microfiber formed at the chalcogenide fiber end. In the final step, the tip of the tapered microfiber was moved close to ceramic microheater, at which time the temperature of the ceramic microheater was raised to 500 °C. Due to the surface tension of the chalcogenide glass, the end of the microfiber melted into a microsphere, thus resulting in a chalcogenide glass microsphere resonator.

**Figure 6:**
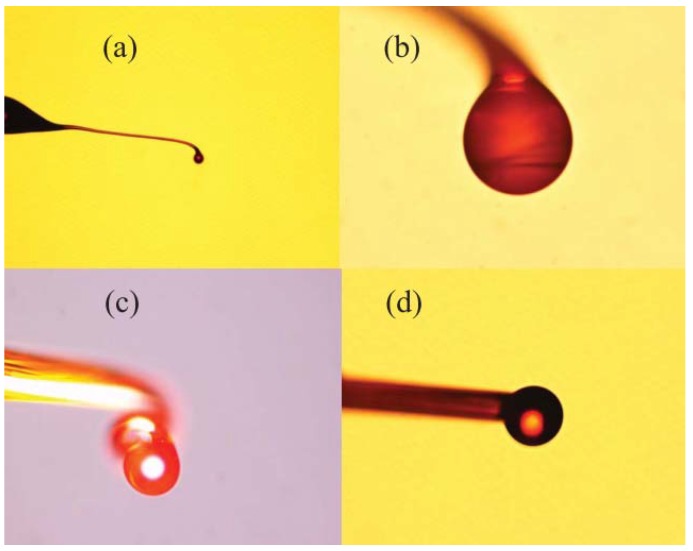
(**a**) Microscope image of the chalcogenide glass microsphere sample with different diameters; (**b**) 53 µm; (**c**) 98 µm; (**d**) 109 µm. Reprinted/Adapted with permission from [70].

Figure 6 shows a microscope image of the chalcogenide microsphere fabricated by the above method: the surface of chalcogenide microsphere appears smooth and uniform.

### 3.5. Fabrication of Microspheres by the Powder Floating Method

The previously mentioned methods for fabricating compound glass microspheres are only capable of producing one microsphere at a time, and the size of the microsphere is determined by the size of the glass optical fiber. Moreover, the method of fabricating the microsphere from the optical fiber introduces additional heating steps during the fabrication process, and this will contaminate the glass material. This is extremely important for chalcogenide glass introduced in the previous section. Therefore, a powder floating method has been proposed [72,73], which is not only capable of producing microspheres of different sizes at the same time, but also effectively avoids the introduction of impurities.

**Figure 7:**
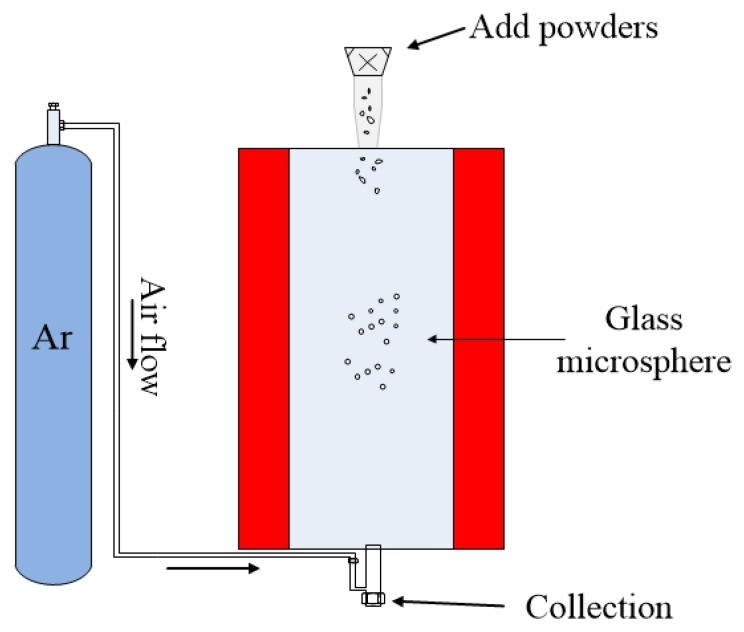
Schematic diagram of fabrication of microsphere by powder floating method.

The schematic diagram of fabrication of microspheres by the powder floating method is shown in Figure 7. During the fabrication process, a sample of compound glass material was ground into powder form, and the powders were allowed to pass through a sieve with openings on the order of tens of micrometers, depending on the required size of the microsphere. Then, the glass powders were dropped from the upper inlet of the furnace. Due to the inert gas (usually argon) flowing in the furnace, the microspheres were suspended in the furnace, with the temperature of the melting furnace (up to 1100 °C) set by the nature of the added glass material. In order to make the glass material melt fully in the furnace, the heating time of the powder in the furnace was increased by increasing the length of the heating zone of the furnace. Finally the molten powder forms glass microspheres due to the surface tension, and the smoothness of the microspheres is ensured by long term heating within the furnace. The advantages of the powder floating method is that the glass microspheres with a size distribution in a certain predetermined range can be fabricated in one batch, which greatly improves the efficiency for fabricating microspheres.

## 4. Application and Characterization of Compound Glass Microspheres

A wide variety of applications based on compound glass microsphere resonators have been reported, which include fiber microcavity lasers, nonlinear optics, optical sensors, and quantum optics. Before introducing the application of the compound glass microspheres, the coupling methods of the microsphere resonators are introduced due to their significant role in experimental realization of these devices. There are two main methods for coupling incident light into the microsphere resonators. The first involves the use of tapered fiber coupling. Most tapered fibers are fabricated in silica fiber, which have lower refractive index when compared with most compound microspheres and tend to excite higher order modes in the microspheres [1,74]. The second method involves prism coupling, the input and output coupling being achieved using total internal reflection in a prism whose surface is located close to the microsphere [75,76,77,78]. The main advantage of this is it facilitates greater structural stability.

In this section, compound glass microcavity lasers and Stimulated Brillouin Scattering (SBS) in tellurite glass microspheres are initially reviewed, followed by an introduction to the application of chalcogenide glass-based microspheres in temperature sensing. Finally, high-Q compound glass microsphere resonators are characterized.

### 4.1. Compound Glass Microspheres Lasers

#### 4.1.1. Silica Microsphere Laser

As early as 2003, the California Institute of Technology proposed a microcavity laser based on a silica microsphere [79], by coating Erbium-doped gels on silica microspheres to obtain a microcavity laser with a low threshold of 28 μW.

Before the experiment, a gel solution of tetraethoxysilane (TEOS) and ErNO_3_·5H_2_O was prepared in an acidic mixture. The silica microsphere was then immersed in the gel solution, and was immediately exposed to CO_2_ laser radiation in order to cure the gel on the microsphere surface. The resulting microsphere had a diameter of 50–80 μm, including the gel layer thickness of 1–10 μm.

**Figure 8:**
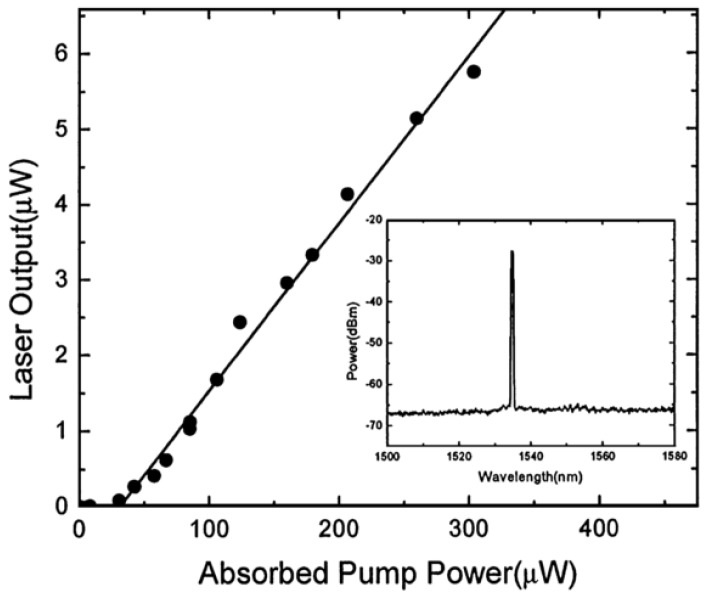
Relationship between the absorbed pump power and laser output; inset: spectrum of the laser output. Reprinted/Adapted with permission from [79].

A single-frequency laser source with a wavelength of 980 nm was coupled to the doped microsphere using a silica tapered fiber with a diameter of 1.6 μm. The WGM transmitted in the microsphere provided the pump energy for the population inversion in the Erbium ions, and generated a single-mode laser output near the wavelength of 1540 nm. The output spectrum is included in Figure 8, which also shows the relationship between the laser output power of silica glass microsphere and the absorbed power; Figure 8 shown that the laser threshold value is 28 μW, and the absorbed power and laser output power are linear.

#### 4.1.2. Phosphate Microsphere Lasers

Phosphate glasses have a higher rare earth ion doping solubility compared to silica. A number of early studies were conducted on the doping of rare earth ions in phosphate glass microspheres. These include Er^3+^, Er^3+^-Yb^3+^ and Er^3+^-Yb^3+^-Cr^3+^ [28,29,31], all of which were successfully made to operate as a microcavity laser. The Er^3+^-Yb^3+^ doped phosphate glass microsphere mentioned in [31] had the lowest laser threshold.

**Figure 9:**
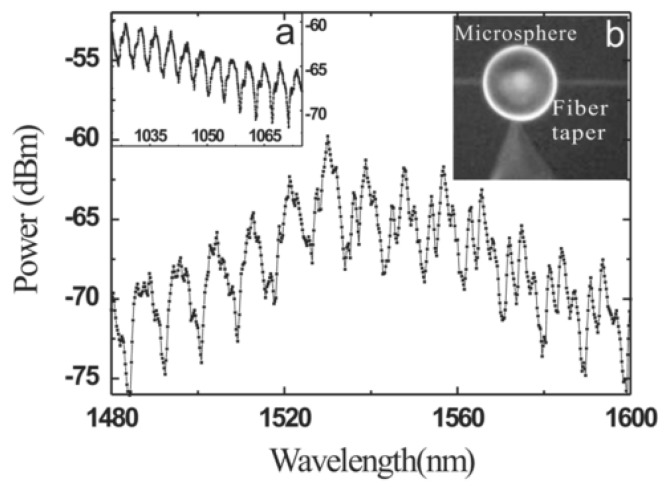
Fluorescence spectra of Er ions doped microsphere. (**a**) The spectrum in the band of 1040 nm; (**b**) the image of the microsphere and tapered fiber. Reprinted/Adapted with permission from [31].

In the case of the microsphere with Er^3+^-Yb^3+^ doped into the phosphate glass microsphere, the diameters of the resulting microsphere and silica tapered fiber were 57 μm and 1.8 μm, respectively. The pump laser was a semiconductor laser with a wavelength of 977 nm. When the excitation power was lower than the laser threshold, the fluorescence spectrum of the Er ions doped microsphere resembled that shown in Figure 9, where the free spectral range (FSR) values at the wavelength region of 1040 nm and 1550 nm are 4 nm and 9 nm, respectively.

**Figure 10:**
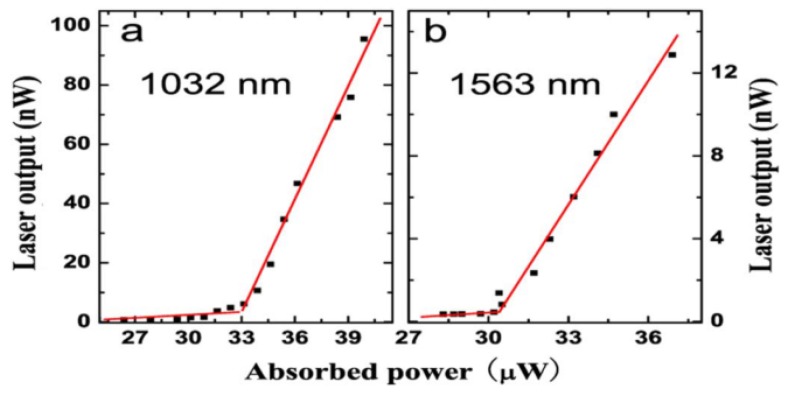
(**a**) Relationship between absorbed power and laser output, the lasing wavelength is 1032 nm; (**b**) the lasing wavelength is 1563 nm. Reprinted/Adapted with permission from [31].

The relationship between the laser output power of the Er^3+^-Yb^3+^ doped microsphere and the absorbed power was obtained by increasing the pump power continuously in a range beyond the laser threshold. As shown in Figure 10, the laser threshold at a wavelength of 1032 nm and 1563 nm are 32 μW and 30 μW, respectively.

#### 4.1.3. Germanate Microsphere Laser

Traditional rare earth ion doped glass microspheres have a specific spectral region in the wavelength range of 1100–1400 nm, where no laser output has been achieved until recently. Bi-doped germanate glass microspheres have been effectively used to compensate for this gap in spectral coverage [37]. Compared to the conventional silica fiber (less than 0.1 mol%), the doping concentration of Bi ions in germanate glass is higher (3.5 mol%), and the melting temperature of the germanate is lower, which is more conducive to the fabrication of Bi doped germanate glass. In addition, the gain coefficient of Bi in germanate glasses is much higher than that in silica, mainly because the phonon relaxation rate in germanate glass (circa 600 cm^−1^) is lower.

**Figure 11:**
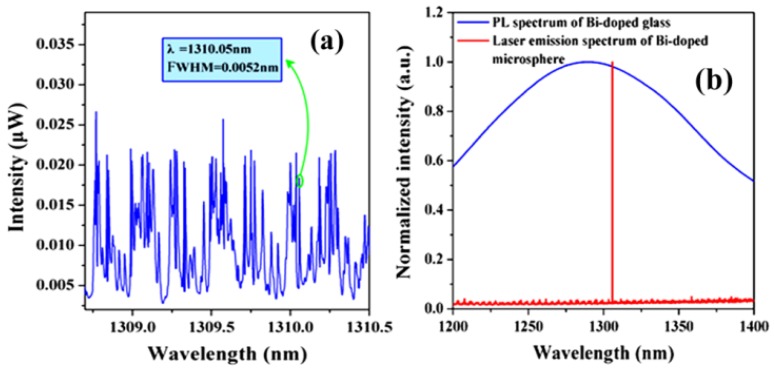
(**a**) Spectrum of the WGMs when the pump light is coupled into the microsphere; (**b**) red line: spectrum of the Bi-doped microsphere laser output; blue line: fluorescence spectrum of the Bi-doped glass. Reprinted/Adapted with permission from [37].

Microcavity lasers based on Bi-doped germanate glasses have been successfully fabricated and are described in the literature [37]. In this experiment, the diameter of the silica tapered coupling fiber and the microsphere were 3 μm and 80 μm, respectively. A laser light source with a wavelength of 808 nm was chosen as the pump. The laser output spectrum of the Bi-doped germanate could be observed on an optical spectrum analyzer (OSA), and is shown in the Figure 11. Figure 11a shows a spectrum of the WGMs when the pump light is coupled into the Bi-doped germanate glass microsphere; the full width half maximum (FWHM) is 0.0052 nm at 1310.05 nm, resulting in a Q value of the Bi-doped glass microsphere up to 2.5 × 10^5^. When the pump absorption power is reaches 215 μW, the output of the single-mode laser at the wavelength of 1305.8 nm was observed, as shown by the red line in Figure 11b. The blue line in Figure 11b is the fluorescence spectrum of the Bi-doped germanate glass.

#### 4.1.4. Tellurite Microsphere Laser

**Figure 12:**
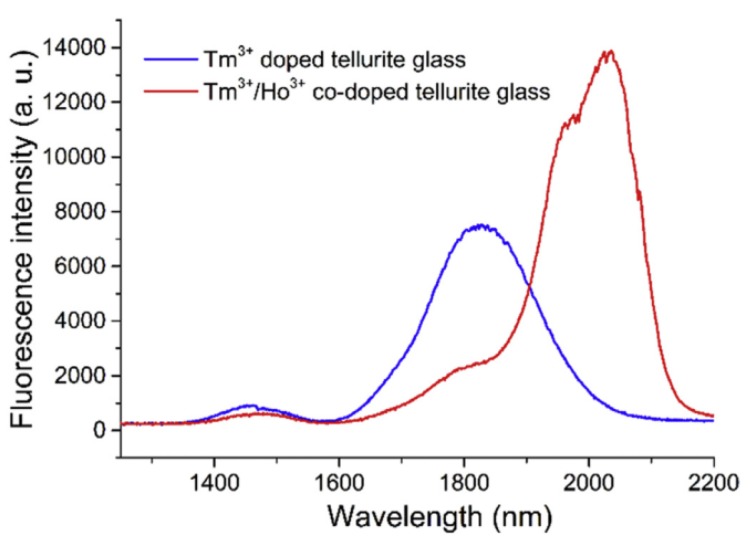
Fluorescence spectrum of the Tm^3+^ doped and Tm-Ho co-doped tellurite glasses. Reprinted/Adapted with permission from [81].

Tellurite glass has emerged as a promising material for use in the MIR wavelength region [80]. In this section, a Tm-Ho co-doped tellurite microcavity laser in the MIR region is introduced [81], with the underlying principle of operation being the transition of the ^3^H_6_ to ^3^H_4_ energy level in Tm^3+^ ions. This was achieved using a pump with a wavelength of 808 nm, resulting in the transition from ^3^H_4_ to ^3^F_4_ energy level in some Tm^3+^ ions, so that the energy in the Tm^3+^ ions could be partially transferred to the adjacent Ho^3+^ ions, prompting the transition of the ions from ^5^I_8_ to ^5^I_7_. Finally, the spontaneous emission of Tm^3+^ ions from ^5^I_7_ to ^5^I_8_ produces a fluorescence emission in the wavelength range 1.8–2.2 μm. The fluorescence spectra of Tm^3+^ doped tellurite glass and Tm-Ho co-doped tellurite glass are shown in Figure 12.

**Figure 13:**
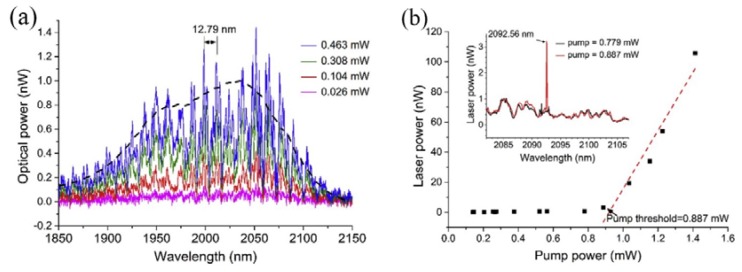
(**a**) Spectrum of WGMs in the tellurite glass microsphere; (**b**) relationship between the output laser power and pump power; inset: spectral output when the pump is below (blue line) and above threshold (red line). Reprinted/Adapted with permission from [81].

In this experiment, the diameters of the silica tapered fiber and the tellurite glass microsphere were 1.38 μm and 59.52 μm, respectively. A laser diode with a wavelength of 808 nm was used to pump the microsphere laser. When the pump power was lower than the threshold value, the fluorescence spectrum was observed using the OSA, and the resulting spectrum is shown in Figure 13a. Each peak of the fluorescence spectrum represents a mode transmitted in the microsphere and the observed FSR of the WGM was 12.79 nm. The black line in Figure 11a represents the fluorescence spectrum of the tellurite glass. However, when the pump power exceeded 0.887 mW, a single-mode laser output was observed from the Tm-Ho doped tellurite glass microsphere. The output wavelength of the single-mode laser was observed to be around 2.1 μm. Figure 13b shows the relationship between the output laser power of the tellurite glass microsphere and the absorbed power. It is clear from the inset in the figure that when the pump power is 0.779 mW or lower, there is no laser output emission, and when the power was increased to 0.887 mW, a single-mode laser output was generated as shown in the inset of Figure 11b, with a center wavelength of the output laser at 2092.56 nm.

### 4.2. SBS in Tellurite Microsphere

Stimulated Brillion Scattering (SBS) is a non-linear optical phenomenon, caused by the electromagnetic stretching effect generated by high-power incident light which stimulates an ultrasonic wave in the waveguide, and results in the incident light being scattered by the ultrasonic waves.

**Figure 14:**
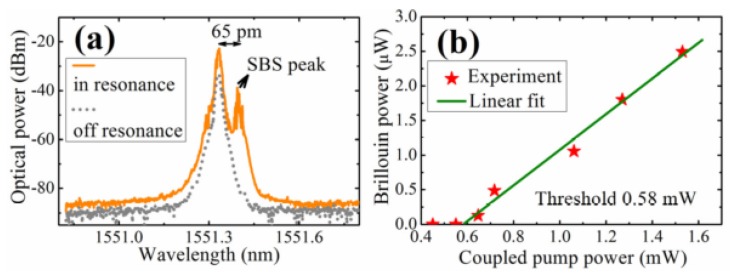
(**a**) Gray line: pump off resonance; yellow line: in resonance; (**b**) relationship between coupled pump power and Brillouin power. Reprinted/Adapted with permission from [82].

In the literature [82], the SBS phenomenon has been reported for the first time in a tellurite glass microsphere, and the Q value of the fabricated microsphere exceeded 1.3 × 10^7^. In the experiment, a tellurite glass microsphere was fabricated using melting methods using a CO_2_ laser. The diameter of the tapered fiber and microsphere were 2 μm and 116 μm, respectively, and a single-frequency tunable laser with a wavelength of 1550 nm was used to pump the tellurite microsphere. The resulting spectrum is shown in Figure 14a, where the gray line shows the spectrum when the pump was off resonance and the yellow line shows the case when in resonance. It can be clearly seen from the spectrum that the SBS phenomenon is generated and observed at a wavelength that is 65 pm higher than the pump peak wavelength, and this represents the first order Stokes signal. Beyond that, the peak intensity of the yellow line is generally 10 dB larger than the peak intensity of the gray line, and the SBS peak of the yellow line is 15 dB larger than the gray line. These results suggest that most of the power in the strong Rayleigh scattering is feedback, thus inhibiting SBS generation. When the pump power was increased from 0.4 mW to 1.6 mW, the relationship of the coupled pump power and the Brillouin output power was obtained, as shown in Figure 14b, resulting in a threshold for the Brillouin output power of 0.58 mW. 

### 4.3. Temperature Sensing Using Chalcogenide Glass Microspheres

The basic properties of chalcogenide glass have been introduced in Section 2.3. Chalcogenide glass has a high photosensitivity, and good transmittance (low attenuation) in the MIR region, making chalcogenide glass microspheres highly sensitive to environmental parameters such as temperature.

In this section, a temperature sensor using chalcogenide glass microspheres in the MIR region is introduced [71]. A Tm^3+^ doped chalcogenide glass microsphere was prepared using the previously mentioned powder floating method. The experimental principle is that the Tm^3+^ transition from the ground state ^3^H_6_ to ^3^H_4_ occurred, followed by the spontaneous transition of Tm^3+^ from ^3^H_4_ to ^3^F_4_, and ^3^F_4_ to ^3^H_6_ generating a fluorescence emission in the 1.5 μm and 1.9 μm wavelength bands.

**Figure 15:**
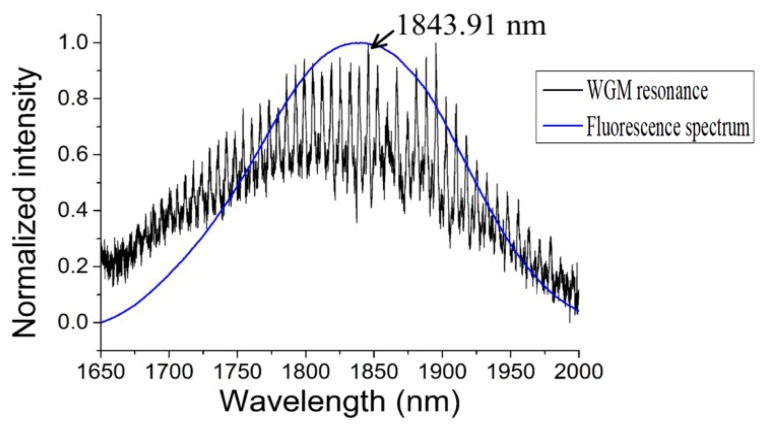
Whispering-gallery mode (WGM) resonance in a chalcogenide glass microsphere (black), compared with the unmodulated fluorescence spectrum of the chalcogenide glass (blue). Reprinted/Adapted with permission from [71].

In the experiment, the diameter of the silica tapered fiber and the chalcogenide glass microsphere were 1.72 μm and 108.52 μm, respectively; pumping was provided from a laser diode with a wavelength of 808 nm. The WGM of the chalcogenide glass microsphere in the wavelength range 1.65 μm to 2 μm was obtained by coupling the pump light into the microsphere, and the spectral output is shown in Figure 15. The blue line is the unmodulated fluorescence spectrum of the chalcogenide glass.

**Figure 16:**
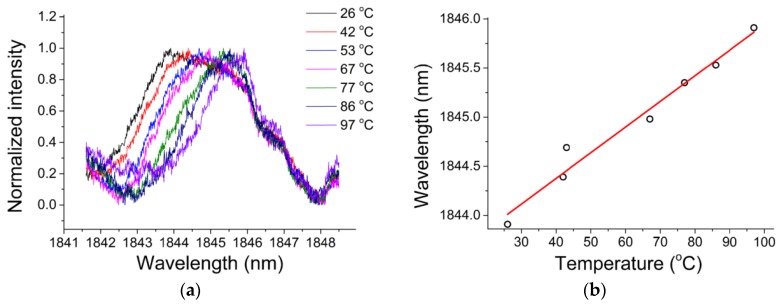
(**a**) Resonance spectra at different temperatures; (**b**) relationship between the wavelength shift and temperature. Reprinted/Adapted with permission from [71].

In order to study the sensitivity of the chalcogenide microsphere to temperature, the whole device was placed in an adiabatic environment, and the ambient temperature was increased from 26 °C to 97 °C, resulting in all the resonances in the spectrum shifting to longer wavelengths. For ease of observation, a resonant peak with a wavelength of 1843.91 nm was chosen as a reference. The deviation of the wavelength of the single resonant peak with the change of temperature is shown in Figure 16a, and the relationship between the wavelength shift and temperature in Figure 16b. A linear relationship exists between the observed wavelength shift and temperature, and the resulting temperature sensitivity of the chalcogenide glass was measured to be 26 pm/°C.

### 4.4. Other High-Q Compound Glass Microspheres

Compound glass doped glass microspheres (such as lead silicate and bismuth glasses) possess a high nonlinear coefficient [64,74].

**Figure 17:**
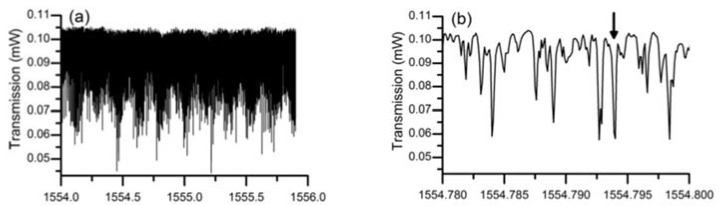

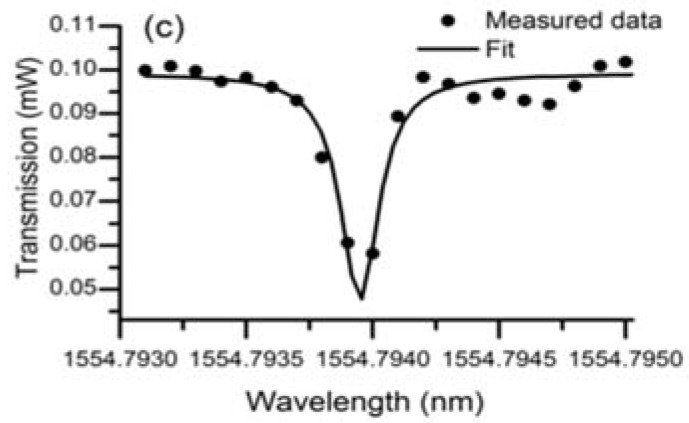
(**a**) Transmission spectra of the lead silicate microsphere in different spectral regions; (**b**) close up spectrum range from 1554.780-1554.800; (**c**) Lorenz fitting curve. Reprinted/Adapted with permission from [64].

In the experiment conducted to characterize the lead silicate glass microsphere, the diameters of the lead silicate glass microsphere and silica tapered fiber were 109 μm and 2 μm, respectively. A pump source with a wavelength of 1550 nm was coupled into the tapered fiber, and the spectrum of the WGM was achieved in the microsphere by effective coupling of the tapered fiber and the microsphere. Finally, the transmitted light from the fiber was received by a photodetector, and the transmission spectrum is shown in Figure 17. Because the effective refractive index of the tapered fiber and the microsphere are quite different, so many high-order modes exist in the transmission spectrum. A wavelength range of 1554.75–1554.8 nm is selected in the spectrum, and the data points obtained in the experiment are fitted using Lorentz fit; the Q value in the microsphere is calculated to be 0.86 × 10^7^.

**Figure 18:**
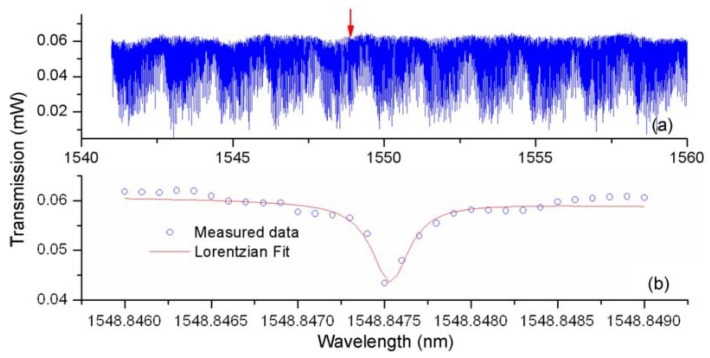
(**a**) Transmission spectra of the bismuth glass microsphere; (**b**) close-up spectrum range from 1548.846–1548.849. Reprinted/Adapted with permission from [74].

The method of characterizing the bismuth glass microsphere is similar to that used for the lead silicate glass microsphere, and is not repeated in detail. The transmission spectrum of bismuth glass microsphere is shown in Figure 18, within the wavelength range from 1548.846 nm to 1548.849 nm, resulting in a Q value of up to 0.6 × 10^7^.

## 5. Conclusions and Outlook

In this paper, a comprehensive review of the properties of compound glasses has been undertaken with a focus on their applications in microsphere devices and sensors. Materials included conventional oxide glasses, heavy metal oxide glasses, and chalcogenide glasses. From their properties, it can be concluded that compound glasses have higher Q, higher rare earth ion doping, and lower phonon energy. The fabrication methods of microspheres using the various compound glass were also reviewed. The traditional method of microsphere fabrication involves melting the glass, which includes fabrication using a CO_2_ laser or a ceramic microheater. For the chalcogenide glass fiber with a low melting point, the microsphere can be directly drawn by the “pulling up” method, as well as the more recently discovered powder floating method, the latter method having the advantage that it allows for mass production of higher quality microspheres. The applications of compound glass microspheres were also studied and these include mainly rare earth ion doped microspheres for lasers, high nonlinearity components involving tellurite glass microspheres, and temperature sensing using chalcogenide glass microspheres.

Compound glass microsphere resonators overcome the limitations associated with traditional resonators in terms of glass materials. In the future, it is envisaged that compound glass microsphere resonators will have wide ranging applications in photonics including optical computers and optomechanics due to their high nonlinearity, high Q quality, and high response. Meanwhile, doping rare earth ions in different host materials is expected to achieve higher power output and more efficient lasers accessing different wavelength ranges, most notably in the infrared band. However, most compound glass-based microspheres are coupled using a tapered fiber, whereas prism-based coupling is more conducive to robust packaging. In general, it is fair to state that the prospects for practical application of compound glass microsphere resonators in photonics are very bright and are developing rapidly.
